# Easy and Efficient Recovery of EMIMCl from Cellulose Solutions by Addition of Acetic Acid and the Transition from the Original Ionic Liquid to an Eutectic Mixture

**DOI:** 10.3390/molecules27030987

**Published:** 2022-02-01

**Authors:** Huan Zhang, Andreea Ionita, Pilar F. Seriñan, María Luisa Ferrer, María A. Rodríguez, Aitana Tamayo, Fausto Rubio Alons, Francisco del Monte, María C. Gutiérrez

**Affiliations:** 1 Instituto de Ciencia de Materiales de Madrid-ICMM, Consejo Superior de Investigaciones Científicas-CSIC, Campus de Cantoblanco, 28049 Madrid, Spain; huanzhang0107@163.com (H.Z.); andreeai@ucm.es (A.I.); pilar.serinan@csic.es (P.F.S.); delmonte@icmm.csic.es (F.d.M.); 2 Area de Cristalografía y Mineralogía, Facultad de Ciencias, Universidad de Extremadura, 06006 Badajoz, Spain; marodgon@unex.es; 3 Instituto de Cerámica y Vidrio-ICV, Consejo Superior de Investigaciones Científicas-CSIC, Campus de Cantoblanco, 28049 Madrid, Spain; aitanath@icv.csic.es (A.T.); frubio@icv.csic.es (F.R.A.)

**Keywords:** ionic liquids, deep eutectic solvents, cellulose dissolution, cellulose regeneration, ionic liquid recovery, cellulose acetylation

## Abstract

Ionic liquids (ILs) and deep eutectic solvents (DESs) are the two most widely used neoteric solvents. Recently, our group described how the simple addition of acetic acid (AcOH) to 1-Ethyl-3-methylimidazolium chloride (EMIMCl) could promote the transition from the original IL to an eutectic mixture of EMIMCl and AcOH. Herein, we studied how cellulose regeneration and EMIMCl recovery from EMIMCl solutions of cellulose could be benefited by the significant differences existing between EMIMCl- and EMIMCl·AcOH-based mixtures and the easy switching from one to the other. Finally, we also demonstrated that the transition could also be accomplished by addition of acetic anhydride and water so that the process could be eventually useful for the achievement of highly acetylated cellulose.

## 1. Introduction

Both ionic liquids (ILs) and deep eutectic solvents (DESs) have become the two most popular neoteric solvents. ILs were originally conceived as molten salts combining a single organic cation and a single organic or inorganic anion so that the charges are neutralized [1]. More recently, certain mixtures of ILs have resulted in the formation of “new solutions” with properties that were somehow different from those of the original ILs [2]. DESs were first described by Abbot and coworkers in 2003 [3] as supramolecular complexes formed between one hydrogen bond donor—HBD—and one hydrogen bond acceptor—HBA, typically an ammonium or phosphonium salt—with the charge delocalization that occurs between the HBD and the HBA being responsible of the decrease in the melting point of the mixture as compared with those of its individual components.

Along the subsequent years of ILs and DESs discovery, few attempts were made to correlate DESs and ILs [4]. More recently, this view has changed thanks to studies describing the presence of H bonds in ILs and aqueous dilutions of ILs with the anion (chloride in most of cases, but other ones may be too) playing a critical role [5,6,7,8,9,10], similarly to what happens in DESs and DESs dilutions [11,12,13,14,15]. In fact, aqueous dilutions of hydrated salts at a certain range of dilution have also been recently described as DESs [16]. Moreover, recent works have described the preparation of mixtures of 1-Ethyl-3-methylimidazolium chloride (EMIMCl, playing the role of HBA) and succinonitrile [17], several amides [18,19] and glycol-derivatives [20,21,22], and different azole [23,24,25] and pyridinium [26] bases, playing the role of HBDs. Most of these DESs, as well as some others based on different imidazolium salts such as 1-butyl-3-methylimidazolium chloride and bromide (BMIMCl and BMIMBr), or 1-hexyl-3-methylimidazolium chloride (HMIMCl [17,27]), were used for gas absorption (mainly SO_2_, but also CO_2_ or NH_3_), and insights about the HB complex structure were obtained by NMR and FTIR spectroscopies as well as by theoretical calculations. In addition, very recent studies performed in our group by NMR spectroscopy and molecular dynamics simulations provided a more detailed description of the HBs formed between EMIMCl and acetic acid (AcOH as the HBD) that allowed one eutectic mixture to be achieved [28]. Interestingly, this work also suggested the possibility of forming this sort of eutectic mixture with different acids as the HBDs (e.g., formic and octanoic acids) as well as with different EMIM-based ILs (e.g., EMIMTFSI).

Herein, we study how the stoichiometric addition of AcOH to a EMIMCl solution of cellulose allows an easy and efficient recovery of EMIMCl thanks to the different solvent properties of EMIMCl- and EMIMCl·AcOH-based mixtures resulting after AcOH addition. Moreover, we design a process to obtain acetylated cellulose in which the transition from EMIMCl- and EMIMCl·AcOH-based mixtures was promoted by addition an efficient acetylation agent such as acetic anhydride (Ac_2_O) instead of the above-mentioned AcOH. The study of EMIMCl recovery and cellulose regeneration either in the non-acetylated or acetylated form was performed by FTIR and NMR spectroscopy.

## 2. Results and Discussion

Based on the specific physicochemical features described elsewhere for EMIMCl- and EMIMCl·AcOH-based mixtures [28], their solvent properties should also be different. There are many papers on dissolving cellulose in the ionic liquid 1-ethyl-3-methylimidazolium acetate (EMIMOAc), which is a very good cellulose solvent (>20 wt% at 80 °C) [29,30]. Moreover, it is well known EMIMCl is an excellent solvent for cellulose [31]. Recent works using molecular dynamics simulations revealed the critical role played by the chloride anion that penetrates between cellulose molecular chains and forms hydrogen bonds with them [32]. The replacement of the original hydrogen bonds between chains promotes their cleavage and ultimately results in cellulose dissolution. Besides the solvent capability, the subsequent cellulose coagulation and IL recovery also accounts on the greenness and economic viability of an IL process for biomass processing. Anti-solvents such as water, ethanol, methanol, acetone, acetonitrile, etc., have been widely used for this purpose, as in the seminal work by Swatloski et al., first describing the cellulose dissolution in ILs [33,34]. Among these solvents, water is chosen in most cases because it is safe, environmentally benign, and inexpensive. However, as regeneration of cellulose occurs when contacting the cellulose solution with the aqueous coagulation bath, the polymer profile at the point of precipitation exhibits a very high interfacial concentration, thus favoring the formation of a dense polymer “skin”, through which further coagulant has to diffuse for bulk sample precipitation. Considering also the competition between the water–anion, cellulose–anion, cellulose–water, cellulose–cation, and anion–cation interactions, full IL separation from cellulose typically requires more than one washing cycle. Based on this, recovering IL for reuse is currently energy intensive—e.g., first involving extensive washing steps with water to recover the IL entrapped within the regenerated cellulose and then intensive evaporative steps for separating the water from the IL—and ultimately compromises the greenness of the whole process. Thus, as stated in a recent review on this field, the development of more efficient and energy-saving methods for the recycling of ILs is required [35].

Interestingly, where both EMIMCl and EMIMOAc are excellent solvents for cellulose dissolution [32,36,37], cellulose solubility is negligible in EMIMCl·AcOH-based mixures, most likely because the strong participation of chloride anions in H bond complexes forming the DES makes them basically unavailable to form new H bonds with cellulose chains. Thus, we hypothesized a process for cellulose regeneration where, after cellulose dissolution in EMIMCl, transition from EMIMCl to EMIMCl·AcOH upon addition of AcOH would result in cellulose precipitation and separation from the EMIMCl liquid phase, without the addition of any antisolvent.

**Figure 1:**
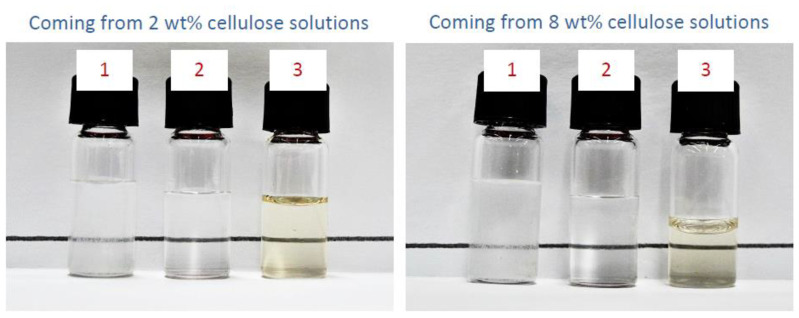
Picture of the supernatant phase obtained after addition of H_2_O at 20 °C (1), H_2_O at 4–5 °C (2), or AcOH at either 20 or 60 °C (3) to EMIMCl solutions with 2 wt% (**left**) and 8 wt% (**right**) cellulose contents.

For comparison, we first performed cellulose regeneration using bare water as the anti-solvent (see entries #1 and #2 in Table 1). In this case, cellulose was precipitated by H_2_O addition (e.g., 10 mL) and gentle stirring over 2 h. The experiment was carried out at 2 different temperatures (e.g., 20 and 4 °C) and on 2 different EMIMCl solutions (e.g., with cellulose contents of 2 and 8 wt%). After centrifugation, cellulose was concentrated at the bottom of the centrifuge tube and separated from the supernatant. The precipitate was subsequently washed with further H_2_O (e.g., 10 mL), centrifuged, and separated from the supernatant, and this process repeated 3 more times (e.g., 10 mL in each) for a total of 5 washing cycles, following a similar process to those described previously [38,39]. Cellulose recovery was accomplished by simple drying of remaining H_2_O, while EMIMCl recovery was accomplished by submission of the supernatant aqueous phase to a freeze-drying process. When H_2_O addition and washing was carried out at 20 °C, cellulose recovered as precipitate was quite poor (ca. 80%). Actually, this poor recovery was anticipated by the cloudy aspect of the supernatant phase (Figure 1), that revealed the presence of some cellulose yet dispersed in the liquid phase. Data of EMIMCl recovery shown in Table 1 (entry #2) should be taken with caution as the presence of cellulose in the supernatant phase resulted in the ultimate overestimation of any figure coming from this experiment. Cellulose recoveries above 95% (near full recoveries, within the experimental error) were obtained when the process was carried at 4 °C and we obtained a fully transparent supernatant phase (Figure 1). EMIMCl recovery was ca. 71% after the 1st washing cycle and we needed 4 additional washing cycles (and the freeze-drying of the supernatant phases, that is, ca. 50 mL) to basically get full EMIMCl recovery (ca. 98%).

Based on the possibility for transitioning from EMIMCl- to EMIMCl·AcOH-based mixtures upon addition of AcOH and the negligible cellulose dissolution in these latter mixtures, we slightly modified the cellulose regeneration process trying to avoid or at least reduce the use of H_2_O for cellulose precipitation and separation from the EMIMCl liquid phase. Thus, we added AcOH (e.g., 10 mL) to the EMIMCl solution of cellulose so that cellulose precipitated upon the transition from EMIMCl- to EMIMCl·AcOH-based mixtures. The clear aspect of the supernatant phase obtained in this case (Figure 1) anticipated an excellent cellulose recovery (e.g., ca. 100%), regardless of whether the process was carried out at 20 °C. Meanwhile, the boiling point of AcOH (ca. 118 °C) allowed its removal from EMIMCl·AcOH-based mixtures by thermal treatment at 60 °C under air flow (Figure S1). Thus, the application of this treatment to the supernatant resulted in AcOH evaporation and an EMIMCl recovery above 60% (Table 1). Interestingly, AcOH could also be added stoichiometrically (e.g., 1, 2, or 3 equivalents to obtain EMIMCl·1HOAc, EMIMCl·2HOAc, or EMIMCl·3HOAc, respectively). In these cases, the process was carried out at 60 °C to decrease the viscosity of the mixture and allow for a better homogenization. Differences in the amount of recovered EMIMCl coming from first precipitation upon the use of different temperatures, amount of AcOH (e.g., 1, 2, or 3 equivalents, or 10 mL) and cellulose contents in solution (e.g., 2 or 8 wt%) indeed confirmed the role played by viscosity in the recovery process (Table 1). The process continued washing the precipitated cellulose with successive additions of H_2_O. Interestingly, EMIMCl recovery increased to ca. 90% after the 1st washing cycle (e.g., with 10 mL), largely improving the recovery yield obtained without the aid of EMIMCl- to EMIMCl·AcOH-based DESs transition (ca. 71%). Full EMIMCl recovery (ca. 99%) was obtained with only two additional washing cycles (Table 1).

**Figure 2:**
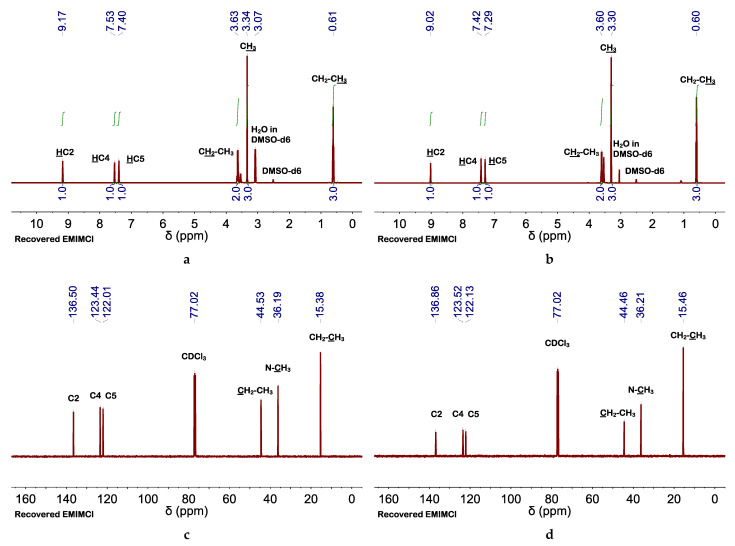
(**a**,**b**) ^1^H and (**c**,**d**) ^13^C NMR spectra of recovered EMIMCl (**a**,**c**) using H_2_O as the antisolvent or (**b**,**d**) adding first AcOH and promoting the transition from EMIMCl- to EMIMCl·HOAc-based mixtures. ^1^H and ^13^C NMR spectra were performed at 85 and 25 °C, respectively.

**Figure 3:**
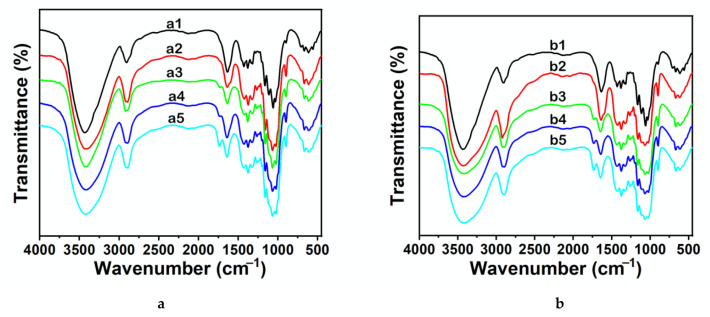
FTIR spectra of cellulose regenerated from EMIMCl solutions. The cellulose contents were 2 wt% (**a**) and 8 wt% (**b**). The antisolvent used for cellulose precipitation was either water (10 mL) at 20 °C (a2, b2, red line) or 1 equivalent of AcOH (a3, b3, green line), 2 equivalents of AcOH (a4, b4, blue line), or 3 equivalents of AcOH (a5, b5, light blue line) at 60 °C. The spectrum of MCC was included in both graphics for comparison (a1, b1, black line).

^1^H NMR spectra of recovered EMIMCl (from both processes, without or with the aid of AcOH addition) exhibited the same peaks than the original EMIMCl (Figure 2). The efficient recovery of EMIMCl in its original form open the path to its reuse in subsequent treatment processes, thus helping to make the process economically viable. Meanwhile, regenerated cellulose was characterized by X-ray diffraction (XRD), Raman and FTIR spectroscopy, and thermogravimetric analysis (TGA). Cellulose regeneration from solution typically implies a crystalline transition from its native lattice (cellulose I) to the more thermally stable cellulose II lattice [40]. In our case, regenerated cellulose was amorphous, as revealed by the vanishing in XRD pattern of any crystalline diffraction peak characteristic of microcrystalline cellulose (MCC) and the appearance of a broad scattering peak centered at ca. 30° in 2θ (Figure S2) [41]. Raman spectra of regenerated cellulose showed bands at 380 cm^−1^ assigned to the out of plane breathing of glucose ring, at 896 cm^−1^ assigned to glucose ring deformation, at 1090–1120 and 1160 cm^−1^ assigned to skeletal deformation, at 1315 and 1338–1378 cm^−1^ assigned to C–C-H, C-O–H, and O-C-H bendings, and at 1470 cm^−1^ assigned to CH_2_ bending (Figure S3) [42]. Interestingly, the main difference between regenerated cellulose and the original MCC was in the bands at 380 and 1096 cm^−1^, typically used to determine the cellulose crystallinity [43]. As for recovered EMIMCl, no significant differences were observed between celluloses recovered without or with AcOH addition. Actually, differences in the regenerated cellulose obtained by the use of one process or the other were only observed by FTIR spectroscopy (Figure 3, Figure S4). In both cases, FTIR spectra exhibited the typical bands of MCC at 3337–3362 cm^−1^ assigned to OH stretching, at 2891–2896 cm^−1^ assigned to CH stretching, at 1430 cm^−1^ assigned to CH_2_ deformation, at 1370–1315 cm^−1^ assigned to C–H and O–H deformation cm^−1^, and at 1045 cm^−1^ assigned to C–O–C pyranose ring skeletal vibration, among the most significant [44,45]. However, the FTIR spectra of regenerated cellulose obtained after addition of AcOH exhibited a band at 1742 cm^−1^ of low intensity that revealed the occurrence of partial cellulose acetylation [46,47]. The low degree of substitution (DS) was further confirmed by the insolubility of our partially acetylated cellulose in DMSO, CH_3_Cl, or acetone, these are typical solvents used to measure the DS by NMR spectroscopy [46,47]. Interestingly, this partial acetylation resulted in an enhancement of the thermal stability of cellulose regenerated with AcOH, as compared with that regenerated without AcOH (see TGA in Figure S5).

**Figure 4:**
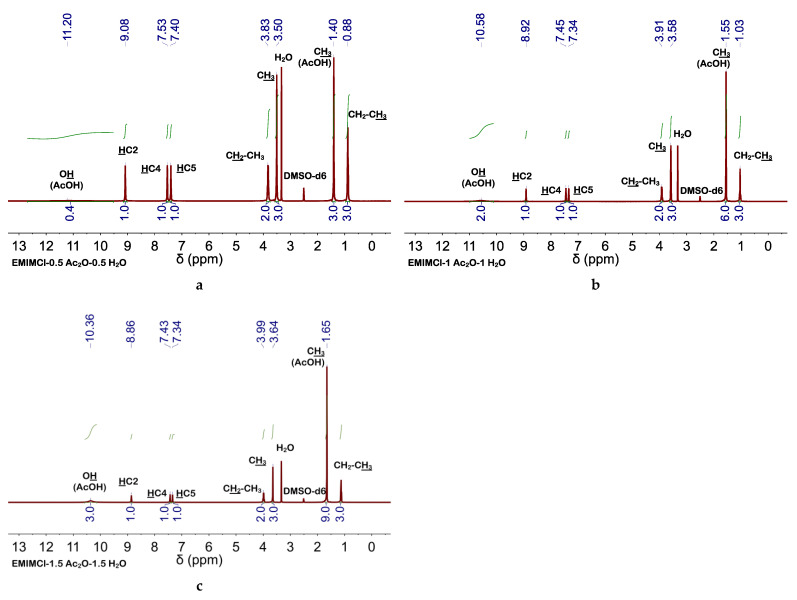
^1^H NMR spectra of EMIMCl·HOAc-based DESs; (**a**) EMIMCl·1HOAc obtained by addition of 0.5 equivalents of Ac_2_O and 0.5 equivalents of H_2_O to 1 equivalent of EMIMCl, (**b**) EMIMCl·2HOAc obtained by addition of 1 equivalent of Ac_2_O and 1 equivalent of H_2_O to 1 equivalent of EMIMCl, and (**c**) EMIMCl·3HOAc obtained by addition of 1.5 equivalents of Ac_2_O and 1.5 equivalents of H_2_O to 1 equivalent of EMIMCl. All ^1^H NMR spectra were performed at 25 °C.

**Figure 5:**
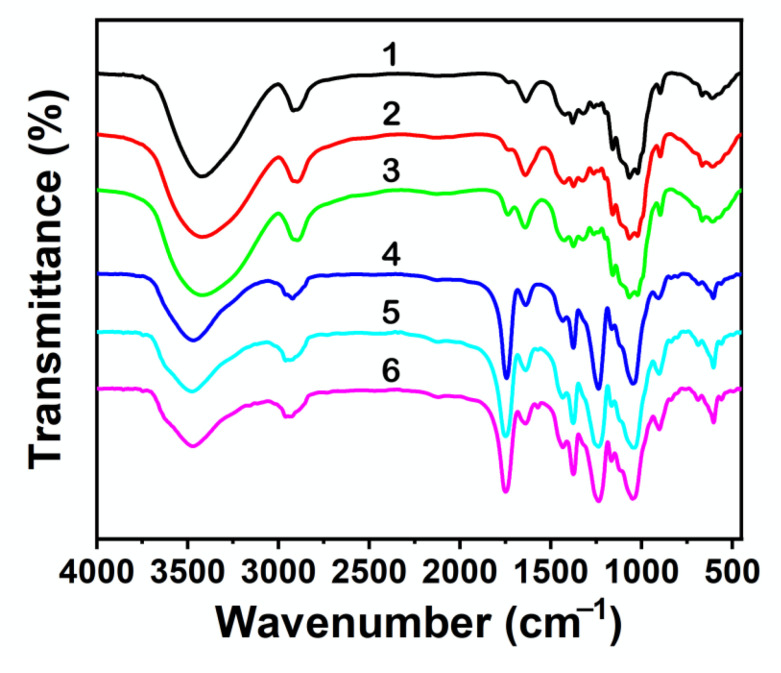
FTIR spectra of cellulose regenerated from EMIMCl solutions with 2 wt% cellulose content. Acetylation was carried out at 60 °C in the presence of 0.5 equivalent of Ac_2_O (4, dark blue line), 1 equivalent of Ac_2_O (5, light blue line), or 1.5 equivalents of Ac_2_O (6, pink line), followed by the addition of 10 mL of H_2_O to promote the EMIMCl- to EMIMCl·HOAc-based DESs transition and cellulose precipitation. The FTIR spectra of precipitated cellulose obtained upon the addition at 60 °C of 1 equivalent of AcOH (1, black line), 2 equivalents of AcOH (2, red line), or 3 equivalents of AcOH (3, green line) were also included for comparison.

**Figure 6:**
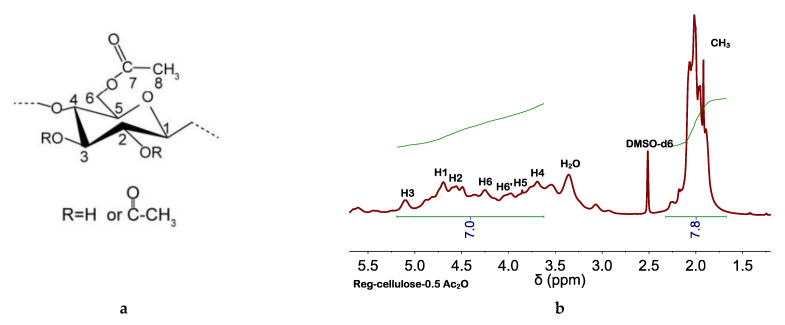

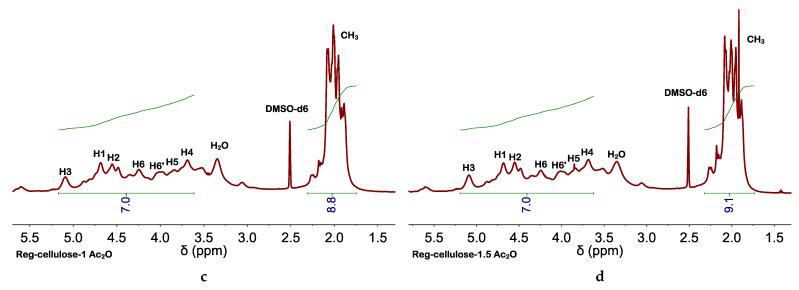
Glucose unit with labelled atoms (**a**) used for the assignment of peaks in the ^1^H NMR spectra of cellulose obtained after acetylation at 60 °C in the presence of (**b**) 0.5, (**c**) 1, or (**d**) 1.5 equivalents of Ac_2_O. DSs of ca. 2.5, 2.9, and 3 were obtained from the integrals of peaks assigned to protons of the acetyl group (CH_3_) and the anhydroglucose unit (AGU)—e.g., $DS=7\times I_{\left(CH_{3},\;H\right)}/3\times I_{\left(AGU,H\right)}$, where 7 and 3 come from the number of protons in the AGU and the number of hydroxyl groups per AGU. All ^1^H NMR spectra were performed at 25 °C.

The occurrence of cellulose acetylation in EMIMCl and EMIMOAc solutions of cellulose has been widely described and high DSs were found with the use of high temperatures (e.g., ca. 100 °C), acid catalysis, and/or acetylating agents (e.g., vinyl acetate, acetic anhydride (Ac_2_O), etc.) [39,48,49,50,51,52,53]. Among these options, we explored two that could be adapted to our process. First one was adding Amberlyst (e.g., a solid acid catalyst that could be separated from the reaction medium by simple filtration) [49] besides AcOH to the EMIMCl solution of cellulose. The presence of Amberlyst favored cellulose acetylation, more so than without using Amberlyst, as revealed by the high intensity of the band at 1742 cm^−1^ (see FTIR spectra in Figure S6), as well as the increase in the thermal stability (see TGA curve in Figure S7). The second option consisted of the Ac_2_O (instead of AcOH) addition to the EMIMCl solution of cellulose. As mentioned above, Ac_2_O is an effective acetylation agent [52,53]. Moreover, transitioning from EMIMCl- to EMIMCl·AcOH-based DESs could also be accomplished in the presence of H_2_O by Ac_2_O hydrolysis (Figure 4). Cellulose acetylation occurred quite efficiently (even more than when using Amberlyst) as revealed by the intensity of the bands at 1742 cm^−1^ in the FTIR spectra, depicted in Figure 5, as well as the DS (e.g., of ca. 2.5, 2.9, and 3 for acetylation with 0.5, 1, and 1.5 equivalents of Ac_2_O, respectively) obtained by NMR spectroscopy (Figure 6, Figure S8), as described elsewhere [54]. TGA curves also revealed the increase in the thermal stability of the acetylated cellulose (Figure S9). Interestingly, cellulose and EMIMCl recoveries were as good as those obtained with AcOH (e.g., in terms of both yields and purity of recovered EMIMCl, see Figure S10).

## 3. Experimental Part

### 3.1. Materials and Methods

1-Ethyl-3-methylimidazolium chloride (EMIMCl) was purchased from Merck (Saint Louis, MO, USA). Acetic acid (AcOH), acetic anhydride (Ac_2_O), microcrystalline cellulose, and Amberlyst were purchased form Sigma-Aldrich (Saint Louis, MO, USA). Ultra-pure water with 18.2 M Ω of resistivity was obtained from an ELGA Maxima Ultra-Pure Water system (ELGA Berkefeld LabWater, Buckinghamshire, UK). All chemicals were used as received without further purification.

### 3.2. Transitioning from EMIMCl to EMIMCl·nAcOH upon the Addition of Either AcOH or Ac_2_O

EMIMCl·*n*AcOH-based DESs could be obtained by simple addition of either AcOH or Ac_2_O. In the former case, details have been described elsewhere [28]. In the latter case, equimolar amounts of Ac_2_O and H_2_O (e.g., 0.5, 1, or 1.5 equivalents of Ac_2_O and H_2_O to obtain DESs with *n* = 1, 2, or 3, respectively) were added to EMIMCl. EMIMCl·*n*AcOH-based DESs were obtained after stirring over 6 h, at 60 °C.

### 3.3. Cellulose and EMIMCl Recovery from EMIMCl Solutions of Cellulose Using H_2_O as the Antisolvent

EMIMCl solutions of cellulose were prepared by dissolving cellulose either 50 or 200 mg of cellulose in 2.5 g of EMIMCl at 85 °C in 30 min to obtain solution with cellulose contents of 2 and 8 wt%, respectively. Cellulose and EMIMCl recovery using H_2_O as the antisolvent was performed by addition of 10 mL of H_2_O to the EMIMCl solutions of cellulose at either 20 or 4–5 °C. The mixture was stirred over 2 h at the selected temperature and then centrifuged. The supernatant was a mixture of EMIMCl and H_2_O, the freeze-drying of which, and subsequent drying at 60 °C overnight, allowed EMIMCl recovery. The precipitate was cellulose with some remaining EMIMCl that was full recovered after successive washing processes with 10 mL H_2_O (up to 4). The washings (e.g., ca. 40 mL) were first freeze-dried, merged with previous fractions of recovered EMIMCl, and then dried at 60 °C overnight for full EMIMCl recovery. The precipitate was also dried at 60 °C for full cellulose recovery.

### 3.4. Cellulose and EMIMCl Recovery from EMIMCl Solutions of Cellulose Using AcOH as the Antisolvent

For AcOH-assisted cellulose and EMIMCl recovery, AcOH (e.g., either 1-2-3 equivalents, or 10 mL) was added to the EMIMCl solutions of cellulose. The mixtures were stirred at either 20 or 60 °C, over 2 h, and then centrifuged. The supernatant was EMIMCl-*n*AcOH (*n* = 1, 2, 3 or the equivalent to 10 mL, ca. 10), the drying of which at 60 °C overnight provided EMIMCl. The precipitate was cellulose with some remaining EMIMCl-*n*AcOH that was full recovered after successive washing processes with 10 mL H_2_O (up to 2). Waters used for washing (e.g., ca. 20 mL) were first freeze-dried, merged with previous fractions of recovered EMIMCl, and then dried at 60 °C overnight for determination of the amount of recovered EMIMCl and characterization. The precipitate was also dried at 60 °C for determination of the amount of recovered cellulose and characterization.

### 3.5. Cellulose Acetylation in EMIMCl Solutions of Cellulose Using H_2_O as the Antisolvent and with the Aid of AcOH as the Acetylating Agent and Amberlyst as the Catalyst

The process was carried out as described above for cellulose recovery using H_2_O as the antisolvent and with the aid of AcOH, but in the presence of Amberlyst (e.g., 2 wt%), at 85 °C and stirred over 2 h. The recovered products (e.g., acetylated cellulose and EMIMCl) were dried at 60 °C prior to characterization.

### 3.6. Cellulose Acetylation in EMIMCl Solutions of Cellulose Using H_2_O as the Antisolvent and with the Aid of Ac_2_O as the Acetylating Agent

The process was carried out as described above for cellulose recovery using H_2_O as the antisolvent, but changing AcOH by equimolar amounts of Ac_2_O and H_2_O (e.g., either: 0.5 equivalents of Ac_2_O and 0.5 equivalents of H_2_O instead of 1 equivalent of AcOH; 1 equivalent of Ac_2_O, and 1 equivalent of H_2_O, instead of 2 equivalents of AcOH; 1.5 equivalents of Ac_2_O and 1.5 equivalents of H_2_O, instead of 3 equivalents of AcOH). The mixture was stirred at 60 °C, over 2 h. After addition of H_2_O (e.g., 10 mL), stirring of the mixture at 60 °C, over 1 h, and subsequent centrifugation, acetylated cellulose was recovered as the precipitate and EMIMCl·*n*AcOH-based DESs (*n* = 1, 2, or 3) were recovered in the supernatant. Full EMIMCl recovery was accomplished by merging of the different fractions obtained after successive washings of acetylated cellulose as described above. The recovered products (e.g., acetylated cellulose and EMIMCl) were dried at 60 °C prior to characterization.

### 3.7. Sample Characterization

^1^H and ^13^C NMR spectra were recorded using a Bruker Avance DRX500 spectrometer (Spectrospin AG, Faellanden, Switzerland) operating at 500 MHz with a 30° pulse, acquisition time of 3.1719 s, relaxation delay of 1 s and 16 scans, and 125.77 MHz, acquisition time of 1.0912 s, relaxation delay of 2 s, and 128 scans, respectively. The samples were placed in capillary tubes, using deuterated DMSO (DMSO-*d*_6_) as the external reference. Acetylated cellulose samples were dissolved in DMSO-*d*_6_ and placed in 3 mm diameter capillary tubes. The peaks were identified and spectra were processed using the software MestReNova (Mestrelab Research, S.L., Santiago de Compostela, SPAIN). FTIR spectroscopy was performed with a Bruker IFS66v spectrometer (Karlsruhe, Germany). Raman spectra were recorded with a Renishaw inVia Raman microscope (Old Town Wotton-Under-Edge, Gloucestershire, UK) using irradiation at 514 nm (100% laser power, 2 mW power, 10 accumulations, and an exposure time of 50 s). XRD patterns were obtained on a Bruker D8 Advance diffractometer (Rheinstetten, Germany) using Cu-Kα radiation (0.05° step size, 3.5 s counting time). Thermogravimetric analyses (TGAs) were carried out in a TA Instrument TGA Q500 (New Castke, Delaware, USA). Samples were placed in an alumina pan in a sealed furnace and heated at 5 °C min^−1^ from 25 to 350 °C (for EMIMCl-*n*AcOH mixtures) and from 25 to 700 °C (for cellulose) under a N_2_ atmosphere.

## 4. Conclusions

EMIMCl- and EMIMCl·AcOH-based DESs exhibited quite different solvent capabilities for cellulose; that is, cellulose solubility was high in EMIMCl but basically negligible in EMIMCl·AcOH-based DESs. Thus, as compared with processes using bare H_2_O as antisolvent, cellulose and EMIMCl recovery from EMIMCl solutions was improved (mainly in terms of shortening the typically tedious washing cycles to obtain high recovery yields) with the aid of AcOH addition and the transition from EMIMCl- to EMIMCl·AcOH-based DESs. Moreover, acetylated cellulose could also be eventually obtained in high yields when, instead of AcOH, Ac_2_O (an efficient acetylation agent) was used for transitioning from EMIMCl- to EMIMCl·AcOH-based DESs.

## Figures and Tables

**Table 1:** Data of recovered EMIMCl after cellulose precipitation from EMIMCl dilutions with 2 and 8 wt% cellulose contents.

Entry	Cellulose Content in Dilution (wt%)	Antisolvent	Recovered EMIMCl (%)		
			After AcOH Addition	After First H_2_O Addition	After End of Washing Cycles
#1	2	H_2_O (10 mL) at 4–5 °C	--	71 ^b^	97.8 ^c^
#2	2	H_2_O (10 mL) at 20 °C	--	71 ^b^	90.0 ^c^
#3	2	AcOH (10 mL) at 20 °C	62.3 ^a^	91.2 (62.3 ^a^ + 28.9 ^b^)	99.6 ^d^
#4	2	AcOH (10 mL) at 60 °C	63.3 ^a^	92.6 (63.3 ^a^ + 29.3 ^b^)	99.6 ^d^
#5	2	AcOH (1 equivalent) at 60 °C	32.4 ^a^	90.6 (32.4 ^a^ + 58.2 ^b^)	99.1 ^d^
#6	2	AcOH (3 equivalents) at 60 °C	42.8 ^a^	90.2 (42.8 ^a^ + 47.4 ^b^)	98.2 ^d^
#7	8	AcOH (10 mL) at 20 °C	24.9 ^a^	88.8 (24.9 ^a^ + 63.9 ^b^)	98.6 ^d^
#8	8	AcOH (10 mL) at 60 °C	41.5 ^a^	88.2 (41.5 ^a^ + 46.7 ^b^)	99.8 ^d^
#9	8	AcOH (1 equivalent) at 60 °C	6.8 ^a^	85.5 (6.8 ^a^ + 78.7 ^b^)	99.1 ^d^
#10	8	AcOH (3 equivalents) at 60 °C	19.3 ^a^	89.3 (19.3 ^a^ + 70.0 ^b^)	99.0 ^d^

^a^ EMIMCl recovered after AcOH removal by thermal treatment at 60 °C under air flow; ^b^ EMIMCl recovered after H_2_O removal by freeze-drying; ^c^ EMIMCl recovered after 4 additional washing cycles; ^d^ EMIMCl recovered after 2 additional washing cycles.

## Data Availability

Not applicable.

## Supplementary Materials

The following supporting information can be downloaded, Figure S1: TGA curves of EMIMCl, AcOH and EMIMCl HOAc-based mixtures; Figure S2: XRD patterns of regenerated cellulose, Figure S3: Raman spectra of regenerated cellulose, Figure S4: FTIR spectra of regenerated cellulose using H_2_O or AcOH as antisolvent, Figure S5: TGA curves of regenerated cellulose using H_2_O or AcOH as antisolvent, Figure S6: FTIR spectra of regenerated cellulose using AcOH as antisolvent and Amberlyst as catalyst, Figure S7: TGA curves of regenerated cellulose using H_2_O or AcOH as antisolvent and Amberlyst as catalyst, Figure S8: ^13^C NMR spectra of cellulose obtained after acetylation. Figure S9: TGA curves of regenerated cellulose using AcOH or Ac_2_O as antisolvent, Figure S10: ^1^H NMR spectra of EMIMCl recovered after cellulose acetylation

## Sample Availability

Samples of all the compounds described in this work are available from the authors.
