# Supplementary material for: Easy and Efficient Recovery of EMIMCl from Cellulose Solutions by Addition of Acetic Acid and the Transition from the Original Ionic Liquid to an Eutectic Mixture
Source: Molecules. 2022 Feb 1;27(3):987. doi: 10.3390/molecules27030987 (PMC8840234; doi:10.3390/molecules27030987)

Supporting Information

of the paper entitled

Easy and Efficient Recovery of EMIMCl from Cellulose Solutions by Transitioning from the Original Ionic Liquid to an Eutectic Mixture

Huan Zhang,[a] M. Luisa Ferrer,[a,*] María A. Rodríguez,[b] Aitana Tamayo,[c] Fausto Rubio,[c] Francisco del Monte[a] and María C. Gutiérrez[a,*]

[a] *Instituto de Ciencia de Materiales de Madrid-ICMM, Consejo Superior de Investigaciones Científicas-CSIC. Campus de Cantoblanco, 28049-Madrid (Spain),* [b] *Area de Cristalografía y Mineralogía, Facultad de Ciencias. Universidad de Extremadura, 06006-Badajoz (Spain),* [c] *Instituto de Cerámica y Vidrio -ICV, Consejo Superior de Investigaciones Científicas-CSIC. Campus de Cantoblanco, 28049-Madrid (Spain)*

Corresponding Authors

* María C. Gutiérrez, e-mail: mcgutierrez@icmm.csic.es; M. Luisa Ferrer, e-mail: mferrer@icmm.csic.es

Number of pages: 12

Number of figures: 10

Outline:

**Figure S1**	**Page S3**
**Figure S2**	**Page S4**
**Figure S3**	**Page S5**
**Figure S4**	**Page S6**
**Figure S5**	**Page S7**
**Figure S6**	**Page S8**
**Figure S7**	**Page S9**
**Figure S8**	**Page S10**
**Figure S9**	**Page S11**
**Figure S10**	**Page S12**

Figure S1: TGA curves carried out under nitrogen flow (100 mL/min) of EMIMCl (red line), AcOH (black line) and EMIMCl·HOAc-based mixtures with 1 (blue line), 2 (pink line), 3 (green line), and 4 (dark blue line) equivalents of AcOH.

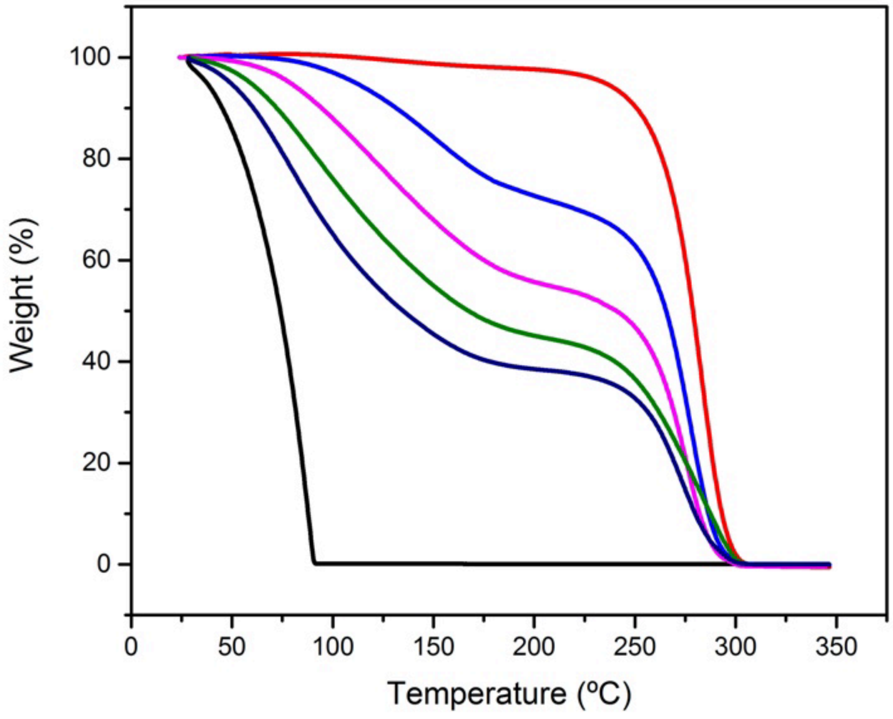

Figure S2: XRD patterns cellulose regenerated from EMIMCl solutions with 2 (red, green, light blue and blue lines) and 8 (pink, orange, dark green and dark blue lines) wt% cellulose. Regeneration was performed with bare water as the antisolvent (red and pink lines) or with the prior addition of 1 (green and orange lines), 2 (blue and dark green lines) or 3 (light blue and dark blue lines) equivalents of AcOH. The XRD pattern of microcrystalline cellulose was included for comparison (black line).

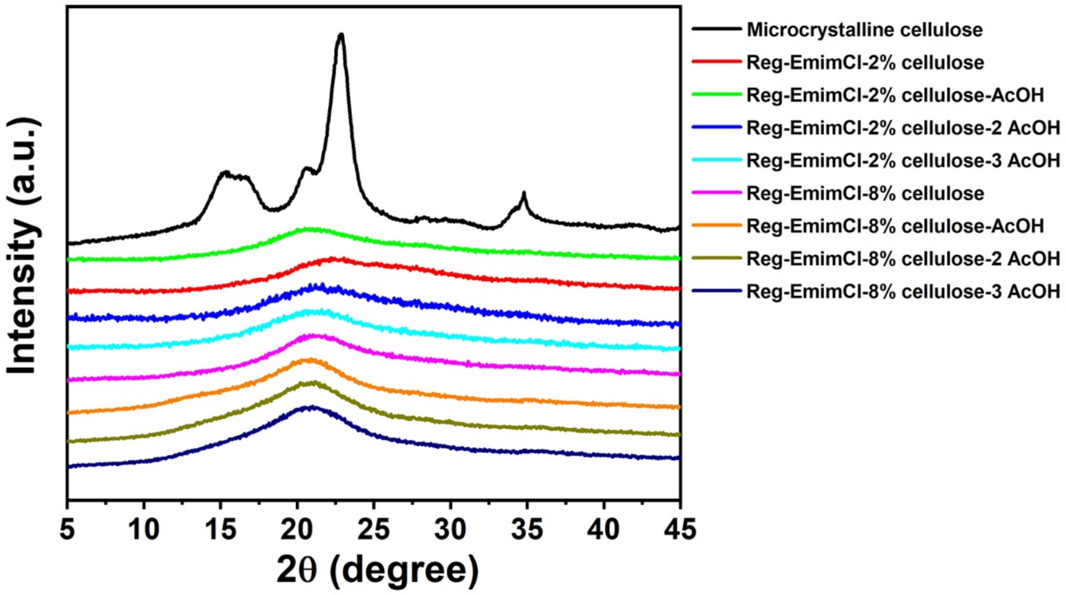

Figure S3: Raman spectra of cellulose regenerated from EMIMCl solutions with (top) 2 and (bottom) 8 wt% cellulose. Regeneration was performed with bare water as the antisolvent (red lines) or with the prior addition of 1 (green lines), 2 (blue lines) or 3 (light blue lines) equivalents of AcOH. The spectrum of microcrystalline cellulose was included for comparison (black lines).

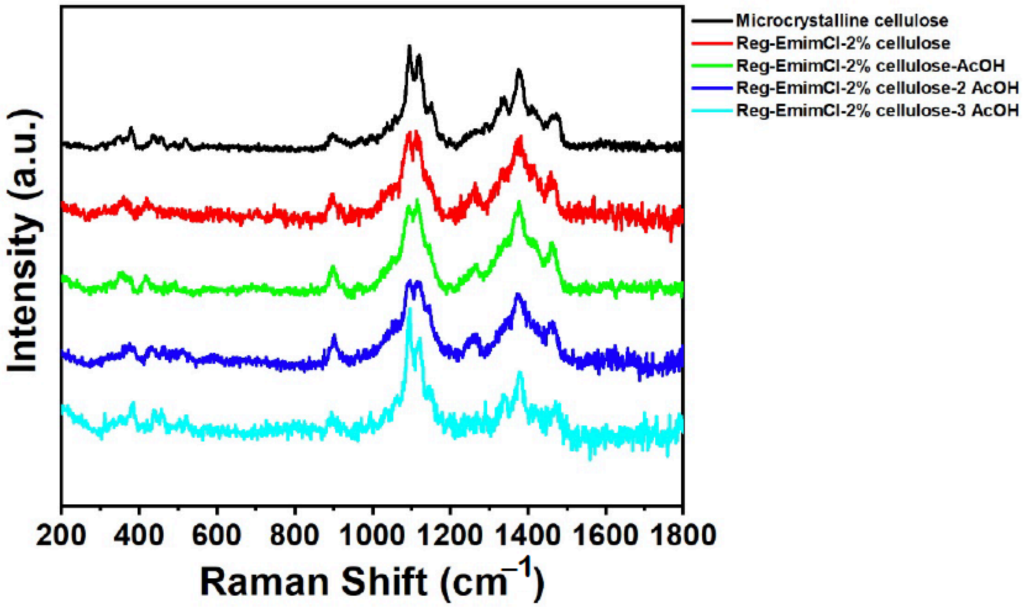

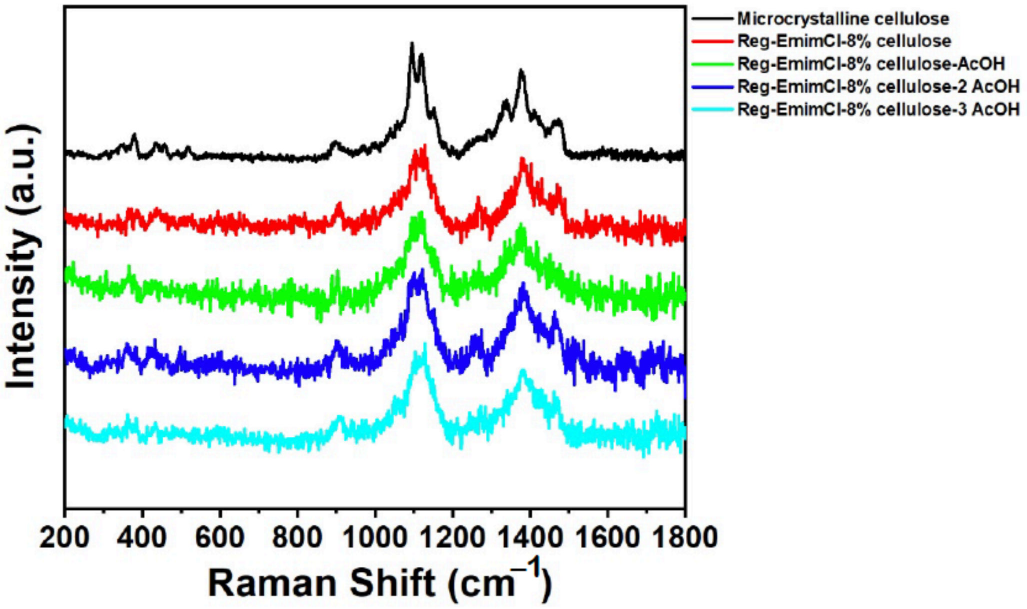

Figure S4: FTIR spectra of cellulose regenerated from EMIMCl solutions with 2 wt% cellulose content. The antisolvent used for cellulose precipitation was either water (10 mL) at 20 °C (red line) or 1 equivalent of AcOH (green line), 2 equivalents of AcOH (blue line), 3 equivalents of AcOH (light blue line) or 10 mL of AcOH (pink line) at 60 °C. The spectrum of microcrystalline cellulose was included for comparison (black line).

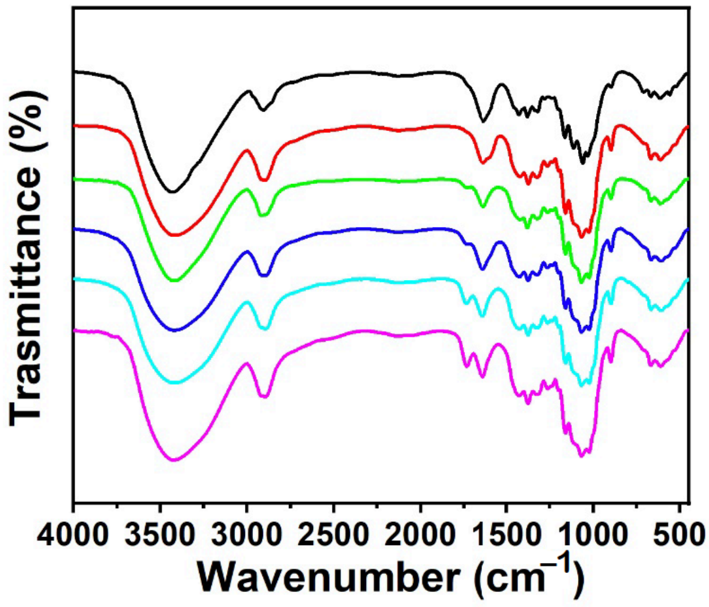

Figure S5: TGA curves of cellulose regenerated from EMIMCl solutions with 2 wt% cellulose content. Regeneration was performed with bare water as the antisolvent (red line) or with the prior addition of 1 (green line) or 3 (light blue line) equivalents of AcOH. The TGA curve of microcrystalline cellulose was included for comparison (black line).

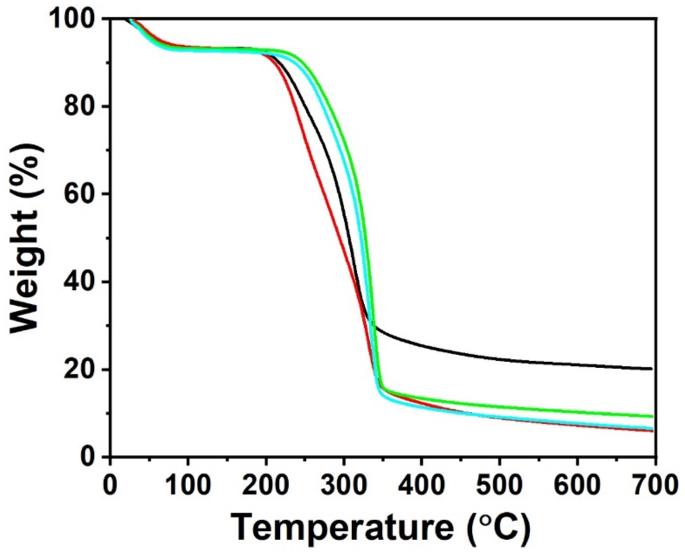

Figure S6: FTIR spectra of cellulose regenerated from EMIMCl solutions with 2 wt% cellulose content. The antisolvent used for cellulose precipitation was 3 equivalents of AcOH, and acetylation was carried out at 60 °C in the absence (light blue line) or in the presence (at 85 °C, grey line) of Amberlyst as the catalyst to promote acetylation.

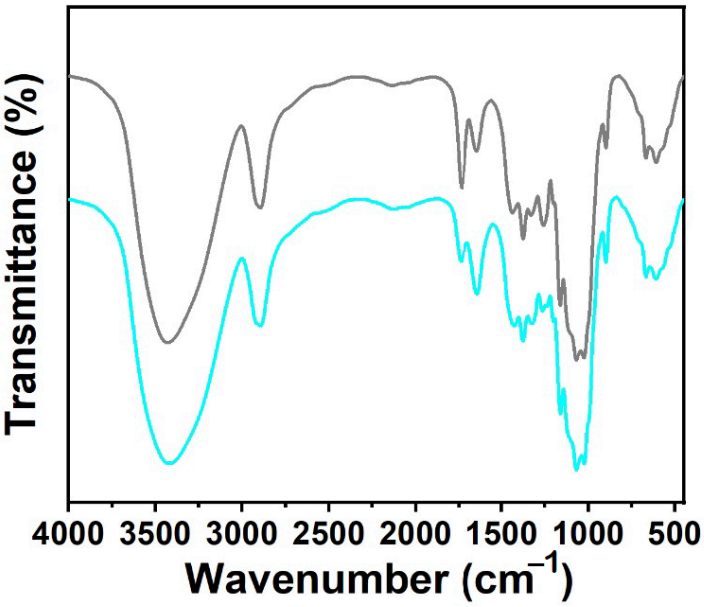

Figure S7: TGA curves of cellulose regenerated from EMIMCl solutions with 2 wt% cellulose content. The antisolvent used for cellulose precipitation was either H_2O (red line) or AcOH (e.g., 3 equivalents), and acetylation was carried out in the absence (at 60 °C, light blue line) or in the presence (at 85 °C, grey line) of Amberlyst as the catalyst. The TGA curve of microcrystalline cellulose was included for comparison (black line).

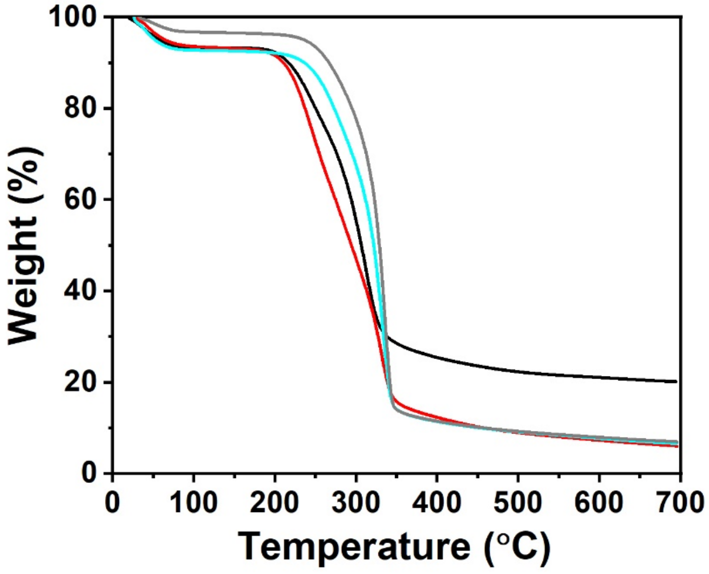

Figure S8: Glucose unit with labelled atoms (a) used for the assignment of peaks in the ^{13}C NMR spectra of cellulose obtained after acetylation at 60 °C in the presence of (b) 0.5, (c) 1 or (d) 1.5 equivalents of Ac_2O. The increase of DS from acetylation with 0.5 to 1 up to 1.5 equivalents of Ac_2O is revealed by the presence of peaks 1 and 1s, and 4 and 4s, being peaks 1 and 4 assigned to carbons next to non-substituted ones (e.g., with hydroxyl groups at C2 and C3, respectively) and peaks 1s and 4s assigned to carbons to substituted ones (e.g., with acetyl groups at C2 and C3, respectively). All ^{13}C NMR spectra were performed at 25 °C.

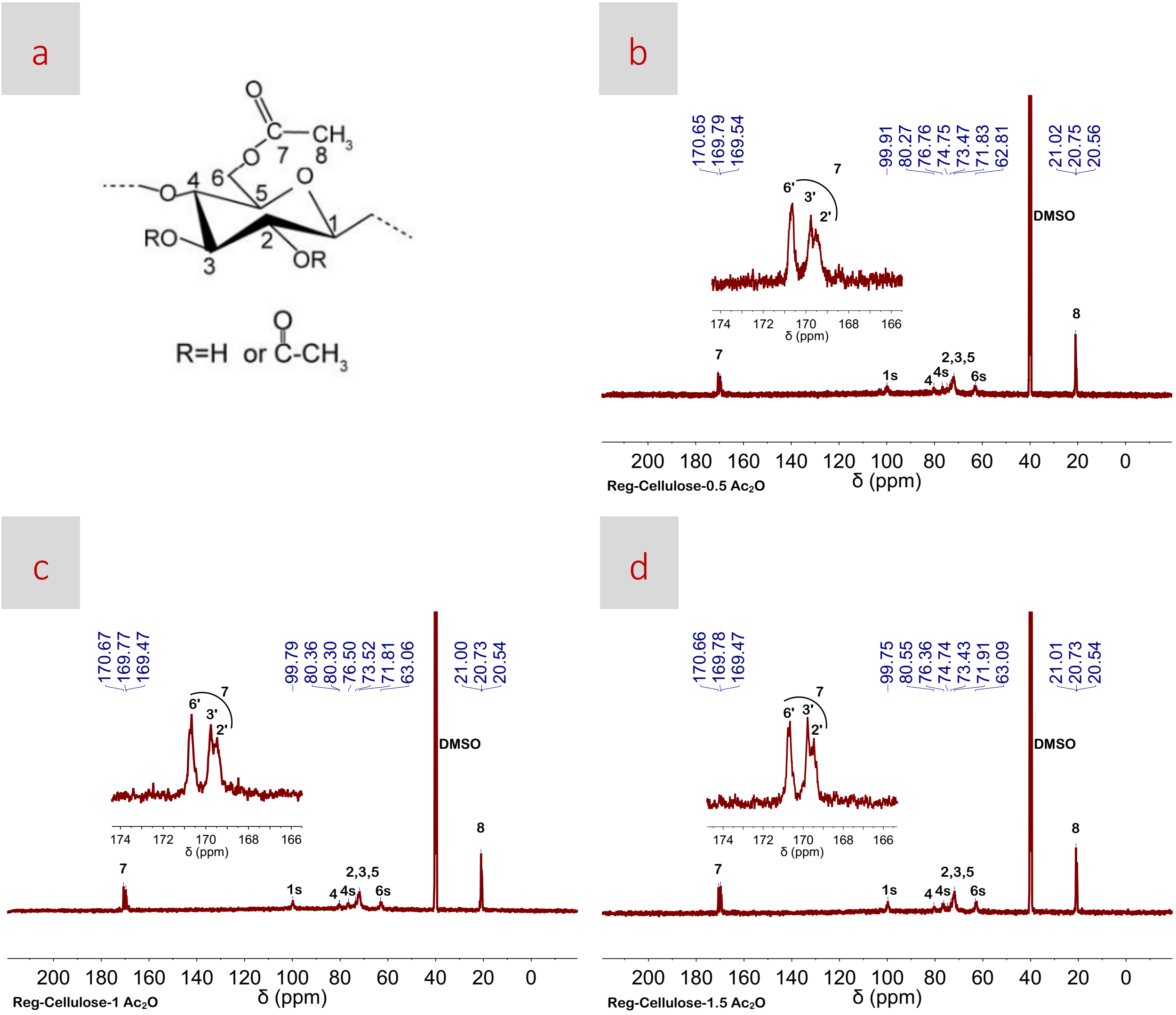

Figure S9: TGA curves of cellulose regenerated from EMIMCl solutions with 2 wt% cellulose content. Cellulose regeneration was accomplished with the addition of 1 (green line) or 3 (light blue line) equivalents of AcOH. Cellulose acetylation was accomplished with the addition of 0.5 (dark green line) or 1.5 (orange line) equivalents of Ac_2O. The TGA curve of microcrystalline cellulose was included for comparison (black line).

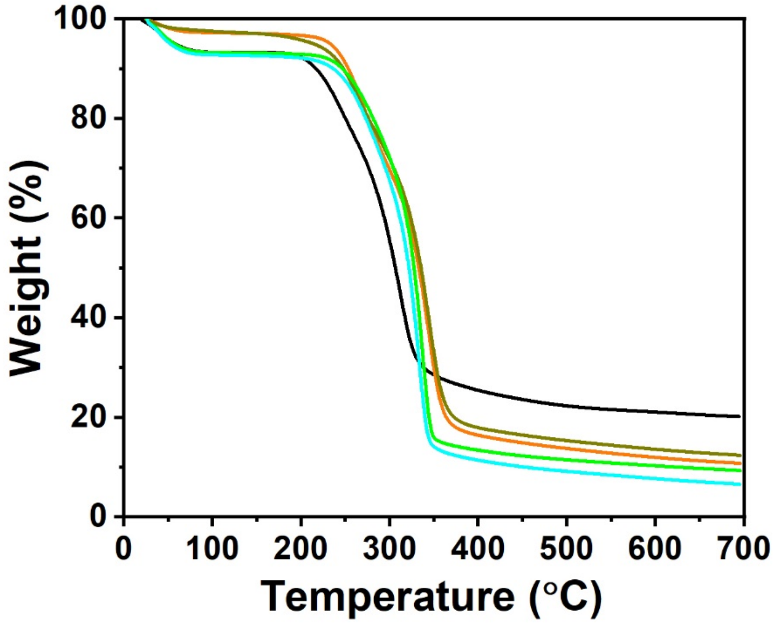

Figure S10: ^{1}H NMR spectra of EMIMCl recovered after cellulose acetylation at 60 °C in the presence of (a) 0.5, (b) 1 and (c) 1.5 equivalents of Ac_2O. All ^{1}H NMR spectra were performed at 25 °C.

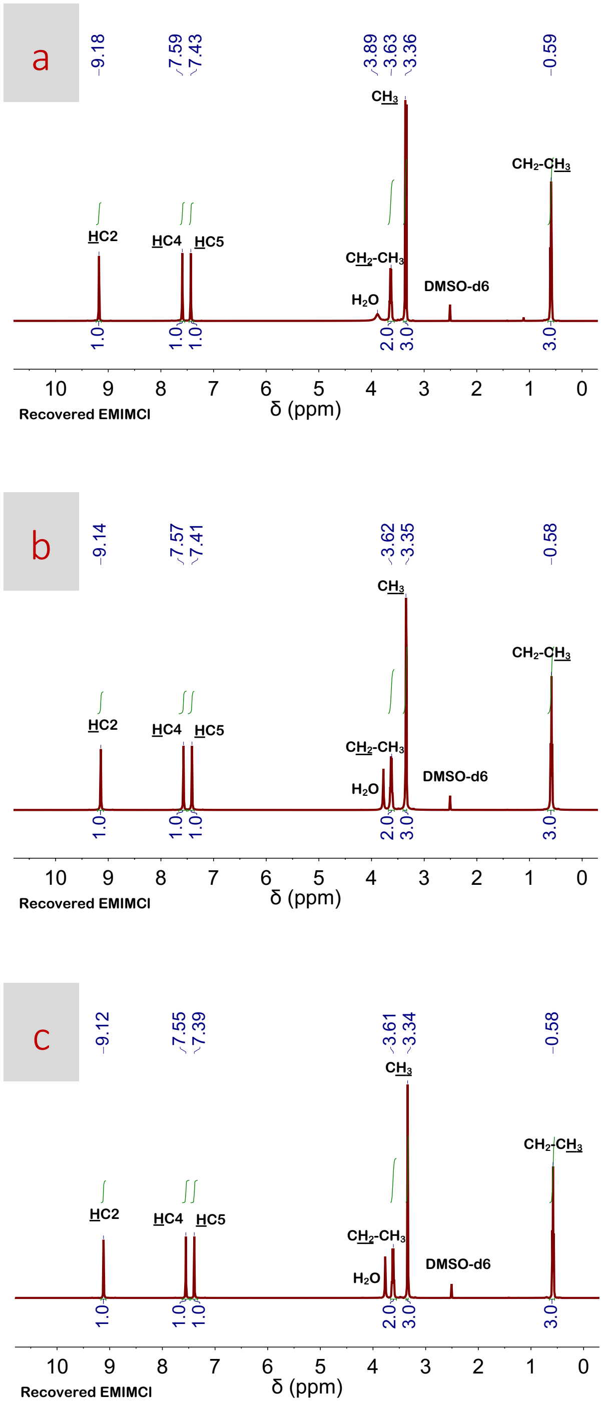

Supplement: Supplementary file 1 [file molecules-27-00987-s001.zip › molecules-1556475-supplementary.pdf]
